# P-1510. The Next-Generation mRNA-1283 COVID-19 Vaccine Elicits Similar and Durable Cellular Immune Responses Compared With mRNA-1273

**DOI:** 10.1093/ofid/ofaf695.1694

**Published:** 2026-01-11

**Authors:** Spyros Chalkias, Bethany Girard, Yamuna D Paila, Wen Zhou, Weiping Deng, Rahnuma Wahid, Md Hasan, Jing Feng, Darin Edwards, Rituparna Das

**Affiliations:** Moderna, Inc., Cambridge, MA; Moderna, Inc., Cambridge, MA; Moderna, Inc., Cambridge, MA; Moderna, Inc., Cambridge, MA; Moderna, Inc., Cambridge, MA; Moderna, Inc., Cambridge, MA; Moderna, Inc., Cambridge, MA; Moderna, Inc., Cambridge, MA; Moderna, Inc., Cambridge, MA; Moderna, Inc., Cambridge, MA

## Abstract

**Background:**

mRNA-1283 is a next-generation COVID-19 vaccine encoding the N-terminal domain (NTD) and receptor binding domain (RBD) of the SARS-CoV-2 spike (S) protein, domains which contain immunodominant epitopes for neutralizing antibody and T-cell responses. mRNA-1283 T-cell responses were evaluated in a phase 2 study.

**Methods:**

Healthy adult participants previously vaccinated with mRNA-1273 received a single 10-µg dose of original mRNA-1283 (encoding original SARS-CoV-2), bivalent mRNA-1283.211 (original SARS-CoV-2 + beta B.1.351), monovalent mRNA-1283.529 (omicron BA.1), or a 50-μg dose of original mRNA-1273 (original SARS-CoV-2) in a phase 2 randomized trial (NCT05137236 ) and were selected to evaluate SARS-CoV-2 S-protein-specific T-cell responses by intracellular cytokine staining . Peripheral blood mononuclear cells were collected at Days 1 (baseline), 29, 181, and 366 and S-specific T-cell responses were measured after stimulation with peptide pools covering the S subunit 1 (S1; comprising RBD, NTD, and subdomains 1 and 2) or S subunit 2 (S2; contains domains not included in mRNA-1283, including C-terminal domain). CD4+ and CD8+ T-cell markers included IFN-γ, TNF-α, and IL-2 (for the type 1 T helper [Th1] response).

**Results:**

Samples from participants who received mRNA-1283 (original, n=29; mRNA-1283.211, n=26; mRNA-1283.529, n=27) and mRNA-1273 (n=21) were included. All participants had detectable T-cell responses at baseline. The frequency of mRNA-1283 S1-specific CD4+and CD8+ Th1 responses increased at Day 29, persisted through Day 366, and were similar to mRNA-1273 for all tested SARS-CoV-2 strains (original, beta B.1.351, and omicron BA.1). CD4+ responses against S2 peptide pool were minimal, as expected based on the S1-targeting design of mRNA-1283.

**Conclusion:**

mRNA-1283 elicited potent CD4+ and CD8+ T-cell responses against SARS-CoV-2 variants using monovalent and bivalent formulations. T-cell responses were similar between mRNA-1283 and mRNA-1273 and persisted for a year after vaccination.

**Disclosures:**

Spyros Chalkias, MD, Moderna, Inc.: Employee|Moderna, Inc.: Stocks/Bonds (Public Company) Bethany Girard, Ph.D., Moderna, Inc.: Employee|Moderna, Inc.: Stocks/Bonds (Public Company) Yamuna D. Paila, PhD, Moderna, Inc.: Employee|Moderna, Inc.: Stocks/Bonds (Public Company) Wen Zhou, PhD, Moderna, Inc.: Employee|Moderna, Inc.: Stocks/Bonds (Public Company) Weiping Deng, PhD, Moderna, Inc.: Employee|Moderna, Inc.: Stocks/Bonds (Public Company) Rahnuma Wahid, PhD, Moderna, Inc.: Employee|Moderna, Inc.: Stocks/Bonds (Public Company) Md Hasan, PhD, Moderna, Inc.: Employee|Moderna, Inc.: Stocks/Bonds (Public Company) Jing Feng, M.S., Moderna, Inc.: Employee|Moderna, Inc.: Stocks/Bonds (Public Company) Darin Edwards, Ph.D., Moderna, Inc.: Employee|Moderna, Inc.: Stocks/Bonds (Public Company) Rituparna Das, M.D., Moderna, Inc.: Employee|Moderna, Inc.: Stocks/Bonds (Public Company)

